# Stereotactic body radiation therapy for centrally-located lung tumors

**DOI:** 10.3892/ol.2014.1815

**Published:** 2014-01-21

**Authors:** GE SHEN, YING-JIE WANG, WEN-JIANG SHEN, ZHEN-SHAN ZHOU, JUN-LIANG WANG, HONG-GUO SHENG, DA-PENG DONG, MING ZHOU, GANG YANG, QIN-WEN WANG, YANJUN ZENG

**Affiliations:** 1 Department of Radiation Oncology, Affiliated Hospital of Academy of Military Medical Sciences, Beijing 100071, P.R. China; 2 Army Radiation Cancer Center and Department of Radiation Oncology, Air Force General Hospital, Beijing 100036, P.R. China; 3 Biomedical Engineering Center, Beijing University of Technology, Beijing 100022, P.R. China

**Keywords:** stereotactic body radiation therapy, lung tumor, centrally-located

## Abstract

The application of high-dose irradiation to centrally-located lung tumors is generally considered to be of high risk in causing bronchial injury. The aim of the present retrospective study was to investigate the safety and efficacy of stereotactic body radiation therapy (SBRT) for patients with centrally-located lung tumors. In total, 28 patients who underwent SBRT for lung tumors within 2 cm of a major bronchus were retrospectively analyzed. The median total dose prescribed was 45 Gy (range, 36.3–52.5 Gy), the median fraction was 12 (range, 10–15) and the median dose per fraction was 3.6 Gy (range, 3–5 Gy). The median follow-up period for the surviving patients was 14 months (range, 10–41 months). The local control rate of SBRT was 100%, with a complete response (CR) rate of 32.1% (9/28); a partial response (PR) rate of 50% (14/28) and a stable disease (SD) rate of 17.9% (5/28). In total, 15 patients survived and 13 patients succumbed; 11 patients succumbed to tumor progression, one to congestive heart failure and one to a brain hemorrhage. The main side-effects included grade 2 esophagitis (17.9%; 5/28) atelectasis (10.7%; 3/28) and grade 2 late radiation pneumonitis (7.1%; 2/28). Severe late toxicity (≥ grade 3) was not observed in any patient. SBRT is an effective and safe therapy for centrally-located lung tumors.

## Introduction

Stereotactic body radiation therapy (SBRT) has been most frequently applied in the treatment of lung tumors (1–6), including the use of SBRT based on the extracranial γ-knife (1–2), which has gradually become the standard treatment of early-stage non-small cell lung carcinoma (NSCLC).

However, Timmerman *et al* (2006) (7) reported that centrally-located tumor cases have a higher proportion of serious adverse reactions following SBRT. The central area is defined as the bronchial tree, consisting of the carina, the left and right bronchus, the left and right upper lobe bronchus, the right middle lobe bronchus, the lingular lobe bronchus, the left and right lower lobe bronchus and the area extending 2 cm from the bronchial tree. The administered dose in this previous study was 60–66 Gy/3 fractions/1–2 weeks. In total, 28 out of 70 patients succumbed; among them, five patients succumbed to a tumor and six patients succumbed to causes related to the treatment. Of the total patients, 14 exhibited grade 3–5 toxicity. Severe toxicity was observed in 46% of patients with a centrally-located tumor. The study indicated that this radiotherapy scheme should not be used for centrally-located tumors. Therefore, the National Comprehensive Cancer Network (NCCN) guidelines for the treatment of NSCLC, in the years 2010 and 2011, defined the central area as a forbidden region for SBRT treatment. Along with the increase in the clinical observations of SBRT, certain studies have challenged this presumption. Chang *et al* (8) reported that the application of other doses of SBRT was safe and effective for centrally-located tumors. The NCCN guideline of 2012 rescinded the decision to make the central location a forbidden area, and recommended that SBRT may be tried on centrally-located tumors with other dose schedules (8,9), but not the unsafe dose schedule of 54–60 Gy/3 fractions reported by Timmerman *et al* (7). More studies (10–14) have explored the effects and the side-effects of SBRT for centrally-located tumors. The adverse reactions are variable due to the difference in ethnicity, equipment and SBRT dosage. The present study reports the clinical observations when using the body γ-knife and SBRT in the centrally-located tumors of Chinese patients.

## Materials and methods

### Subjects

Between May 2009 and May 2012, a total of 28 patients with 46 tumor lesions were treated by body γ-knife. A total of 22 lesions were located in the hilar region, while 19 mediastinal lesions were 1 cm away from the esophagus and 5 mediastinal lesions were within 1 cm of the esophagus. Of the total lesions, 23 were ≤2 cm, 18 were between 2–5 cm and 5 were ≥5 cm in size. The characteristics of the patients are summarized in Table I, and the pathology, staging and medication are summarized in Table II. In total, 15 patients received first-line chemotherapy, eight patients received second and subsequent lines of chemotherapy and five patients did not receive systemic chemotherapy; a 71-year-old patient refused chemotherapy, three patients were extremely old so were presumed unable to tolerate chemotherapy (≥77 years old) and one patient exhibited idiopathic thrombocytopenia. Written informed consent was obtained from the patients and the study was approved by the Ethics Commitee of the Department of Radiation Oncology, Affiliated Hospital of Academy of Military Medical Sciences (Beijing, China).

### Equipment

The Moon God γ-knife manufactured by Shenzhen ET Medical Group (Futian, Shenzhen, China) was used in the study, with 42 cobalt-60 sources and a dose rate of >2 Gy/min.

### Radiation

Computed tomography (CT) scans were performed for three fractions, including the end of the expiratory phase, the end of the inspiratory phase and the free-breathing phase. The images were transferred to the γ-knife system. The tumor target area and the associated normal tissues were delineated, and the displacement of the tumor was measured in three directions (X, Y and Z) (15). CT venography (CTV) was consistent with the gross tumor volume (GTV). GTV plus the displacement range was the internal target volume (ITV), and the expansion by 2–3 mm in each direction was the planning target volume (PTV). The prescribed dose and fraction are summarized in Table III. The dose line (in the range of 50–60%) covering 95% of the target area was the prescribed dose. The dose schedule was 3–5 Gy for each fraction/10–12 fractions; total 36–50 Gy (1,2,16).

### Follow-up

From the time of the γ-knife therapy, the follow-up continued until the patient succumbed or until October 1, 2012.

### Evaluation of efficacy and toxicity

The efficacy was evaluated according to the Response Evaluation Criteria in Solid Tumors (World Health Organization, 2000) (17). The toxicity was evaluated according to the National Cancer Institute Common Toxicity Criteria, v3.0 (18). Early toxicity was defined as toxicity ≤90 days after the start of therapy. Late toxicity was defined as >90 days after the start of therapy.

### Statistics

Statistical analysis was carried out on multiple factors that affected survival time, using SPSS software (SPSS, Inc., Chicago, IL, USA). The Kaplan-Meier method was used to evaluate the survival rates. The survival durations were evaluated from the day of treatment. The log-rank test was used to compare the different levels of a factor. Cox Regression model was used for multivariate analysis of survival. P< 0.05 is considered to indicate a statistically significant difference.

## Results

### Survival and local control rate

By October 1, 2012, 15 patients remained alive. The median follow-up period was 14 months (range, 10–41 months). The median survival time was calculated as 13 months (range, 4–41 months) from the start of SBRT. There were nine complete response (CR) cases, 14 partial response (PR) cases and five stable disease (SD) cases. CR plus PR accounted for 82.1% (23/28). No recurrent cases occurred in the irradiated region during the follow-up period. Of the 13 patients that succumbed, 11 succumbed to tumor progression, one to heart failure and one to a brain hemorrhage.

**Figure 1:**
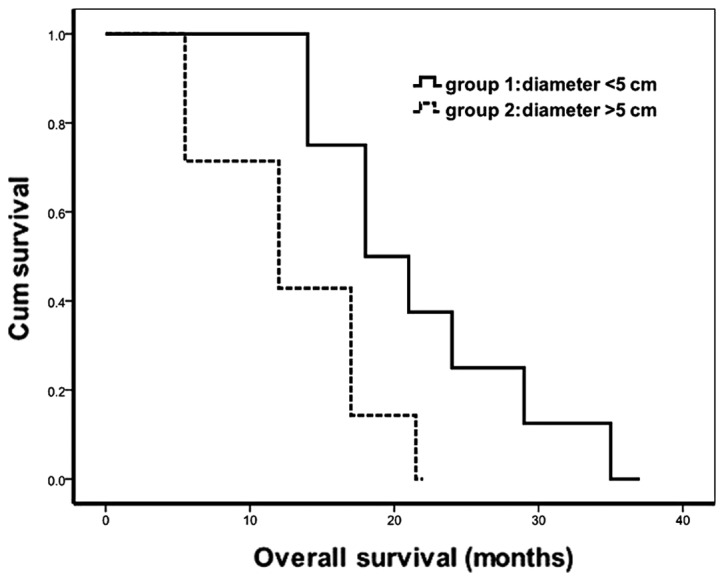
Comparison of the survival time of patients with a tumor diameter of <5 cm (group 1) and >5 cm (group 2).

**Figure 2:**
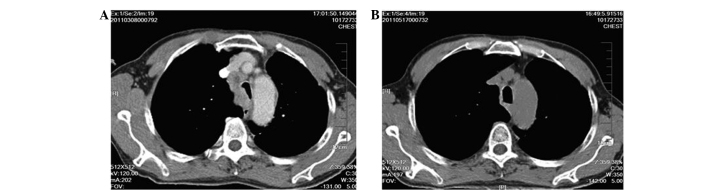
A 70-year-old male patient with squamous carcinoma. (A) Tumor in the main trachea prior to SBRT (2011-03-08). (B) Following SBRT the lesions had completely disappeared with no significant adverse reactions. The patient had not succumbed by the time of analysis (2011-5-17). SBRT, stereotactic body radiation therapy.

Among the 15 cases of NSCLC, 13 patients were male and two patients were female. Nine patients exhibited adenocarcinoma, six patients exhibited squamous cell carcinoma and the median dose of the biologically effective dose [BED_10_, where α/β=10; BED=nd(1+d/α/β)] was 58.8 Gy (range, 48.3–73.8 Gy). In total, seven patients were classified as phase IIIb and eight patients were classified as phase IV. The median age was 64 years old (range, 48–84 years old). The Eastern Cooperative Oncology Group (ECOG) scores were 0 for eight patients, 1 for four patients and 2 for three patients. The tumor diameter was ≤2 cm in three patients, 2–5 cm in five patients and >5 cm in seven patients. The median survival time was 19 months (range, 11–37 months). A total of 9 patients succumbed. The median follow-up period of the 6 surviving patients was 19 months (range, 17–33 months). The tumor size (p=0.028) and ECOG score (p=0.025) significantly affected survival time in the multivariate analysis using the Cox proportional hazards model. Other factors had no significant effect: Stage III vs. stage IV, p=0.812; pathological type (squamous cell carcinoma vs. adenocarcinoma), p=0.717; age, p=0.866; and dose (BED_10_), p=0.928. In the two groups of patients with tumor diameters of <5 cm or >5 cm, the median survival time was 21 months (range, 17–37 months) and 13 months (range, 11–22 months), respectively; log-rank test, p=0.042 (Fig. 1). The tumor of a typical patient is demonstrated in Fig 2.

Of the seven small cell lung carcinoma (SCLC) patients, one patient succumbed. The patient was a 70-year-old male in the extensive stage, with 7 lines of chemotherapy and conventional radiotherapy of the primary lesion prior to SBRT. The patient succumbed to disease progression 4 months after SBRT. The medium follow-up period of the six survivors was 14 months (range, 13–41 months). When these patients received SBRT, three patients were in the extensive stage and three patients were in the limited stage.

### Toxicity

In total, five patients had acute esophagitis; grade 1 in two patients and grade 2 in three patients. All patients completely recovered from this condition following anti-inflammatory and infusion therapy 2 months after radiotherapy. Of the five patients, two survived and three succumbed (two patients succumbed to disease progression and one patient succumbed to heart failure). The tumors were all located ≤1 cm away from the esophagus. The dose was 3 Gy in 15 fractions for three patients, 3.3 Gy in 14 fractions for one patient and 3.6 Gy in 12 fractions for one patient.

Three patients were reported to have atelectasis. The occurrence time was 3.5, 7 and 31 months after irradiation, respectively, and the dose for all three was 3 Gy in 15 fractions. The lobes of two patients were expanded through bronchoscopic treatment of lavage and expansion. By the end of the statistics period, 1 SCLC patient in the limited stage had been followed-up for 40 months and had a good quality of life. One patient with stage IV adenocarcinoma succumbed to lung disease progression; the patient survived for 37 months after irradiation. One patient with stage IIIB adenocarcinoma was reported to exhibit atelectasis 3.5 months after irradiation; the patient refused bronchoscopic lavage and the condition improved following symptomatic treatment.

One of two patients with phase 2 radiation pneumonitis had a history of chronic bronchitis for >10 years. The right hilar, 4R and 7 area lymph nodes were treated by γ-knife at the same time. Telephone follow-ups revealed that the patient’s cough had intensified five months after irradiation, and thus was treated with antibiotics. The other patient was a 58-year-old male with multiple metastases revealed following a right upper lung adenocarcinoma resection; a new lesion (diameter 2 cm) was observed in the right lower lung 3 months after gefitinib treatment. The patient had the first γ-knife radiation treatment and exhibited a CR; the disease progressed 9 months after irradiation. The patient then received GP regimen chemotherapy (1,000 mg/m^2^ gemcitabine on days 1 and 8, and 75 mg/m^2^ cisplatin for the first 3 days) for 4 cycles and paclitaxel single-agent chemotherapy for 3 cycles. A new lesion appeared in the hilum of the right lung (near the tumor first treated by the γ-knife) 9 months after chemotherapy. The second γ-knife therapy was conducted. Telephone follow-ups revealed that grade 2 radiation pneumonitis occurred 3 months after irradiation, and this was treated by antibiotics. The patient succumbed to a brain hemorrhage 5 months after the second SBRT.

## Discussion

With regard to the SBRT dose, Timmerman *et al* (7) reported that the toxicity in centrally-located tumor cases caused by SBRT was 11 fractions of that of the peripheral tumor. The dose was as high as 60–66 Gy/3 fractions, with a BED_10_ of 181–211 Gy and a BED_3_ of 460–550 Gy. Joyner *et al* (2006) (14) studied nine patients with centrally-located lung tumors that were treated by SBRT. The dose was 36 Gy/3 fractions, with a BED_10_ of 79 Gy and a BED_3_ of 180 Gy; grade III tracheal stenosis was observed in one patient. The dose reported by Chang *et al* (2008) (8) was 40–50 Gy/4 fractions, with a BED_10_ of 80–103 Gy and a BED_3_ of 173–258 Gy. No serious adverse reactions were observed in the trachea and the lung. The dose used by Haasbeek *et al* (10) was 60 Gy/8 fractions, with a BED_10_ of 105 Gy and a BED_3_ of 210 Gy; grade III chest pain was reported by two patients, and grade III dyspnoea was observed in two patients. The SBRT dose based on extracranial γ-knife was 3–5 Gy/10–15 fractions (1,2,16). The dose in the present study was 45–51 Gy/10–15 fractions, with 48–75 Gy for BED_10_ and 76–133 Gy for BED_3_. These doses resulted in higher local control rates and the side-effects were acceptable.

Patients with an advanced stage of disease may not necessarily be treated with an excessively high dose. Relatively low dose schedules may be more appropriate for such patients, with mild side-effects and good local control effects within the survival time. For patients with long-term survival, the local control rate and tolerance should be considered when determining the radiation dose for centrally-located tumors. The NCCN guidelines (2012) have recommended that the use of 54–60 Gy/3 fractions is unsafe for centrally-located tumors, but that other dose regimens may be safe and effective. Under the premise of complying with the limit dosage for normal tissues, tumors >5 cm can be treated by SBRT.

Joyner *et al* (14) reported that 36 months after SBRT, certain patients complained of airway stenosis. In the study by Miller *et al* (19), the incidence of airway stenosis was 7% one year after high-dose radiotherapy and 38% four years after high-dose radiotherapy. Oshiro *et al* (11) reported one patient with atelectasis, combined with a severe cough and expectoration, one year after radiotherapy. The symptoms were alleviated after 2 months of repeated balloon dilation therapy. However, Bral *et al* (20) reported that one patient with a centrally-located tumor treated for tracheal stenosis succumbed following the therapy. This indicated that invasive procedures on the trachea are risky following high doses of radiation. In the present study, three patients that reported atelectasis had tumors near the opening of the lobe and segmental bronchi, and there was re-expansion in two patients following bronchoscopic treatment.

Acute esophagitis is associated with the location of the tumor, and the concept of the central area should be classified by the location. SBRT is a precise radiotherapy; the dose of radiation on the adjacent tissues is higher, while the dose received by remote tissues is greatly reduced. The adverse reactions may demonstrate a greater difference with adjacent tissues. Wulf *et al* (21) reported that 3 months after irradiation, mediastinal lesions at 30 Gy/3 fractions and ulcerative esophagitis appeared. In the present study, the patients irradiated due to hilar and front-middle mediastinal lesions did not report acute esophagitis. Of the five patients suffering from acute esophagitis, the distance of the tumor to the esophagus for all was ≤1 cm, and these patients all fully recovered in 2 months. Thus, it is feasible to treat these tumors with a dose of 3 Gy in 15 fractions or 36 Gy in 12 fractions.

In total, two patients suffered from grade 2 late radiation pneumonitis in the present study. One patient had a past history of chronic bronchitis. For the other patient, the tissues irradiated by the second irradiation were close to that of the first irradiation. SBRT should be applied with more caution to patients with poor lung function or a history of chronic lung disease. Oshiro *et al* (11) reported that following treatment of hilar tumors with SBRT in 21 patients, three were observed with dyspnea of grade 3 and above, and two had a history of radiotherapy. Wulf *et al* (21) reported that one patient receiving the second-line radiotherapy had hemoptysis and succumbed. The second SBRT on the adjacent tumor or local tumor should be performed with caution, and the radiation dose should be decreased to reduce the occurrence of side-effects.

In the majority of cases, the doctors performing radiotherapy are faced with similar anatomical site diseases from different primary tumors. Overall survival time is associated with the type of disease, staging and the biological behavior of the primary tumor, and is further associated with systemic therapy and the local treatment of other parts. The difference between the survival time of patients with stage III and stage IV NSCLC was not significant in the present study. Cheruvu *et al* (22) reported 146 cases of NSCLC. The 5-year survival rate of patients with stage III, stage IV and recurrent stage IV, starting from diagnosis, was 7, 14 and 27%, respectively. The 5-year survival rate of stage IV patients with ≤8 lesions was significantly improved in comparison to stage III patients, who developed extensive metastasis and were not suitable for SBRT. The survival rate was 14 and 0%, respectively (P<0.00001).

Wu *et al* (2) studied 43 cases of stage I/II NSCLC treated by SBRT. The median follow-up period was 22 months (range, 3–102 months). The 1- and 2-year local control rate was 75 and 60% for tumors of ≤3 cm, 84 and 71% for tumors of 3–5 cm, 55 and 14.6% for tumors of 5–7 cm and 45 and 21% for tumors of >7 cm, respectively. Clinical staging is an important factor affecting the local control rate (P=0.000) and overall survival (P=0.015). The present study demonstrated that tumor size significantly affected the survival time when SBRT was applied to treat stage III and IV NSCLC.

Patient age had no effect on the survival time. There were three 75-year-old patients, one with phase IIIb NSCLC, one with mediastinal lymph node metastasis of a laryngocarcinoma and one with thymic carcinoma; all survived well following SBRT by the end of the statistical period.

In short, centrally-located lung tumors could be treated by SBRT, with an appropriate dose schedule. In the present study, the local control rate was high and the adverse reactions were well tolerated. The centrally-located area should be subdivided according to the different anatomical sites and the distance from the esophagus. However, more thorough and detailed studies are required.

## Figures and Tables

**Table I:** Characteristics of 28 patients.

Characteristic	Value
Gender, n	
Male	21
Female	7
Age, n	
<65 years	18
≥65 years	10
Median age (range), years	58 (28–84)
ECOG score, n	
0–1	24
2	4
Pathological type, n	
Lung cancer	22
Squamous	6
Adeno	9
SCLC	7
Other tumors	6

ECOG, Eastern Cooperative Oncology Group; SCLC, small cell lung carcinoma.

**Table II:** Pathology, stage and systemic treatment of 28 patients.

Pathology	Stage	Cases, n	No-medication cases, n	First-line, n	Second-line, n	Third-line, n
NSCLC	IIIa	2		2		
	IIIb	5	1 (84-year-old)	4		
	IVa	6	1 (71-year-old refused)	4	1	
	IVb	2	1 (Idiopathic thrombocytopenia)		1	
SCLC	Limited	3		2	1	
	Extensive	4		3		1
Breast cancer	IV	2				2
Colon cancer	IV	1				1
Thymic carcinoma	IV	1	1 (81-year-old)			
Laryngocarcinoma	IV	1	1 (77-year-old)			
Thyroid carcinoma	IV	1			1	

NSCLC, non-small cell lung carcinoma.

**Table III:** Dose and fraction received by 28 patients.

Total dose, Gy	Case, n	Dose, Gy/fraction	Fractions, n	BED_10_, Gy
50	1	5	10	75
51.6	3	4.3	12	73.788
52.5	1	3.5	15	70.875
48	6	4	12	67.2
45	1	4.5	10	65.25
46.2	3	3.3	14	61.446
43.2	4	3.6	12	58.752
45	8	3	15	58.5
36.3	1	3.3	11	48.279

BED_10_, biologically effective dose.
